# Supplementary material for: Survey data and human computation for improved flu tracking
Source: Nat Commun. 2021 Jan 8;12:194. doi: 10.1038/s41467-020-20206-z (PMC7794445; doi:10.1038/s41467-020-20206-z)
Supplement: Supplementary file 1 — Supplementary Information [file 41467_2020_20206_MOESM1_ESM.pdf]

Supplementary Information: Survey Data and Human Computation for Improved Flu Tracking

Stefan Wojcik[1] Avleen Bijral[2] Richard Johnston[2]
Juan M. Lavista Ferres[2] Gary King[3] Ryan Kennedy[4]
Alessandro Vespignani[5] David Lazer[3,5]

[1]Twitter
[2]Microsoft
[3]Harvard University
[4]University of Houston
[5]Northeastern University

December 3, 2020

Contents

1 Supplementary Methods **3**
1.1 Introduction . . . 3
1.2 Sources of Data . . . 3
1.3 Survey Recruitment and Fielding . . . 3
1.3.1 Source Vendor Panel and Sampling . . . 3
1.3.2 Survey Fielding Timeline . . . 4
1.3.3 Survey Questions . . . 6
1.3.4 Survey Recall . . . 6
1.4 Information from Healthcare Providers vs. the Internet . . . 12
1.5 Query and Web Page Labeling . . . 12
1.5.1 Overview of Labeling . . . 12
1.5.2 Labeling Scheme . . . 13
1.5.3 Codebook: Descriptions of Labels . . . 13
1.5.4 Labeling Queries and Pages from Respondent Browsing History . 14
1.6 Query Data Geographic and Temporal Coverage . . . 18
1.6.1 Predicting Internet Behavior Based on Reported Symptoms . . . 20
1.6.2 Demographic Groups and Queries . . . 23
1.6.3 Classifying Flu Cases . . . 27
1.6.4 Robustness Checks: Alternative Panel Models . . . 29
1.6.5 Takeaways from Panel Analysis . . . 30
1.7 ILI Tracking Model . . . 30
1.7.1 Selecting and Labeling Additional Bing Queries . . . 31
1.7.2 Accounting for Demographics: Query Smoothing and Re-weighting 34
1.7.3 More Discussion of Forecasting Results . . . 35
1.7.4 State Level Findings . . . 36
1.7.5 Software and Data . . . 37
1.8 Notes from main paper . . . 37

1 Supplementary Methods

1.1 Introduction

We describe a method for analyzing survey data and search history for real-time tracking of Influenza-like Illness (ILI). This supporting document outlines in detail the steps we took to implement the survey, analyze it, and build a flu-tracking model from it. We first describe the process for labeling queries according to whether they reflect an underlying flu-like experience. We use these labeled searches and survey responses to discern differences between users in the onset of ILI symptoms and flu search behavior. We then use insights from this analysis to build an aggregate-level flu-tracking model.

1.2 Sources of Data

Data from this study come from the following sources:

- Survey data from a panel individual users coupled with Internet search and browsing history
- Census data on demographic distributions at the zip code level (largely from the American Community Survey)
- Daily anonymized search queries at the zip code level from the Bing search engine
- Weekly flu reports from the Centers for Disease Control (CDC)

1.3 Survey Recruitment and Fielding

1.3.1 Source Vendor Panel and Sampling

We partnered with a survey vendor, Luth Research, who works periodically with Microsoft Research conducting opinion research. Their ongoing panel includes approximately 20,000 individuals with personal computers [1]. Luth research recruits users based on a quota system to construct a panel that is representative of Internet-connected US adults. All individuals agreed to participate in marketing research in return for monetary compensation. This agreement included installing a program to track their web browsing and search activities while also responding to questionnaires. All survey and panel data are purged of any personally identifiable information before they are received for analysis.

We selected two subsets from the full 20,000 panel to participate in our research. One set of participants met the following criteria: 1) they had executed queries in any search engine (incl. Bing, Google, Yahoo), 2) these queries included predefined flu-related keywords (e.g. ‘flu’, ‘fever’, ‘influenza’, ‘swollen’, ‘cough’, ‘pneumonia’, ‘sore throat’), or these users visited flu-related URLs (e.g. on WebMD, CDC, Wikipedia). The second

[1] Luth Research conducted recruitment for the panel, adheres to a strict privacy policy, and complies with ISO standards of data security and HIPPA regulations.

group was a comparison group that did not execute a flu-related query or visit a flu-related web site.

Using the 2014-2015 flu season we collected all query data starting November 2014 through February 2015 to use as a benchmark in matching users. Individuals in our sample who made flu-related searches had, on average, much higher search volumes than those who did not. To account for this uneven mixture, we matched users on their search volume quantiles. The dataset was set up into 7 quantiles based on the level of search volumes and non-flu-search cases were paired with flu search cases on a 4:1 basis.

In total we had 1,180 in the search group and 4,000 in the non-search group. After this matching, there was no statistically significant difference in mean search volume between the groups ($p = 0.77$). From these individuals, we collected demographic information and collected survey results from a total of 654 individuals, of which 10 did not have any reported search volume in the sample period (see supplementary table 5). This left us with 262 who had searched a flu-related keyword or site and 382 who did not (omitting the 10 with zero search volume).

Supplementary Table 1: Age distributions in survey invitees and respondents

Age Group	Percent in invitees	Percent in respondents
18-24	2%	5%
25-34	13%	20%
35-44	19%	22%
45-54	23%	21%
55-64	26%	20%
65+	16%	11%

Supplementary Table 2: Gender distributions in survey invitees and respondents

Sex	Percent in invitees	Percent in respondents
Male	40%	39%
Female	60%	61%

1.3.2 Survey Fielding Timeline

We fielded our flu survey in the spring of 2015. We fielded a first wave of the survey from March 19th to March 27th 2015, then followed up with a second wave in the field from April 27th to April 31 to supplement sample size. This field period was largely after the flu season had peaked - the flu season typically peaks in February, but sometimes peaks in December, January, and March. During the 2014-2015 season, the flu peaked

Supplementary Table 3: Flu search terms and Gender among survey invitees

	No flu term	Flu term
Male	68%	32%
Female	54%	46%
Overall	59%	41%

Supplementary Table 4: Flu search terms and Gender among respondents

	No flu term	Flu term
Male	57%	43%
Female	38%	62%
Overall	46%	54%

Supplementary Table 5: Distributions of survey data

Statistic	N	Mean	St. Dev.	Min	Max
Volume	644	5.688	1.610	0.000	9.492
Female	654	0.610	0.488	0	1
Parent	654	0.315	0.465	0	1
Spouse	654	0.509	0.500	0	1
Age	654	4.610	1.434	1	7
Household ILI	654	0.349	0.477	0	1
Respondent ILI	654	0.245	0.430	0	1
Spouse ILI	516	0.174	0.380	0	1
Child ILI	206	0.422	0.495	0	1

in December https://www.cdc.gov/flu/pastseasons/1415season.htm. We collected accompanying browsing data when a user completed the survey. The earliest query or page visit containing key words associated with flu was 10-31-2014 and the latest was 3-31-15.

1.3.3 Survey Questions

Our survey questionnaire asked respondents for demographic, household, and flu-related information. With regard to the flu, we were interested in whether respondents, or a member of their household, recently had a flu-like experience, which we inferred from a combination of self-reported symptoms. We asked respondents about all symptoms of influenza-like illness since November 2015, followed by a question about which month these were experienced (see wording below). We repeated these questions separately for different members of the household, asking about children and other adults in the household (including spouses). Respondents indicating they experienced both fever and cough were labeled as having a flu-like experience (though we also tested our models using a label of fever + cough or fever + sore throat as the label, see section 6.4).

We then followed up by asking which sources[2] (if any) respondents used when seeking information about these symptoms and health care provider diagnoses (if relevant).

With regard to demographics, we asked about respondent gender (male/female/unknown), age, marital status, and number of children in the home. See supplementary table 5 for means and standard errors of survey variables of interest. 'Volume' in the table refers to the logged number of searches by a respondent, and 'ILI' (Influenza-like Illness) is defined as when respondents report both fever and cough for themselves or family members.

We saved the entire browsing history of all panel users, enabling us to examine web page visits and search queries simultaneously - since some users save health pages such as 'WebMD' in their browsers for convenience. Incorporating information about flu-related web visits in addition to queries allows us a more complete picture of user behavior in the presence of flu-like symptoms.

1.3.4 Survey Recall

As we noted in the main text, one potential limitation of this analysis is that we asked respondents to recall symptoms over the course of the flu season. Rubin and Baddeley (1989) describe the problems of recall relating to the length of time between the event and the survey. Memory is more effective for recent events, creating a recency effect. Dating error increases in a linear fashion as period increases - though remains unbiased. In other words, respondents are unlikely to systematically overreport or underreport symptoms, though they are increasingly likely to make recall errors as the time window increases. Another potential issue is boundary effects - where asking respondents whether they experienced flu or cough from November 1, 2014, to the present, they make errors

[2]Health care provider, health website, search engine, book, friend or none

	Household flu	Respondent flu	Spouse flu
(Intercept)	−0.82	−1.05	−1.13
	[0.53; 0.13]	[0.57; 0.07]	[0.74; 0.13]
Volume	0.05	0.10	0.03
	[0.06; 0.38]	[0.06; 0.12]	[0.08; 0.69]
Female	0.24	0.08	−0.45
	[0.19; 0.20]	[0.20; 0.67]	[0.24; 0.06]
Parent	1.34	0.53	0.25
	[0.18; 0.00]	[0.19; 0.01]	[0.24; 0.30]
Age	−0.23	−0.23	−0.19
	[0.07; 0.00]	[0.07; 0.00]	[0.09; 0.03]
Early Response	0.39	0.19	0.51
	[0.24; 0.10]	[0.25; 0.43]	[0.34; 0.13]
AIC	758.25	701.23	471.15
BIC	785.05	728.03	496.52
Log Likelihood	-373.12	-344.61	-229.58
Deviance	746.25	689.23	459.15
Num. obs.	644	644	507

Note that bracketed items below coefficients include [standard error; two-sided unadjusted p-value]

Supplementary Table 6: Rare events logit coefficients estimated using Zelig 5.1.6.1 [7]. Analysis comparing early responders with late responders in reported flu-like symptoms. The outcome variable is self-reported flu-like experience in the household, for the respondent, and for a spouse.

in judgment that can only be later in time than the start and earlier in time than the beginning of the interval - so they may pile up near the center of the interval.

We checked whether respondents who completed the survey closer to the start of the season were more or less likely to report flu symptoms personally or in the household [3]. Since we fielded an initial survey wave from March 19th to March 27th 2015, then a second wave from April 27th to April 31 surveys prior to April 15 2015, we looked at differences between these two survey waves. We did not see differences in the rates that early responders reported symptoms of themselves or the household relative to late responders. We checked whether controlling for respondent timing of submission affected our main result, but it did not substantively alter the coefficient on the household flu variable.

[3]We thank an anonymous reviewer for suggesting this analysis

Survey Question Variables and Wording

This section presents the survey response options and survey questions asked.

Demographics

Gender

- Male
- Female

Age

- below 18
- 18-24
- 25-34
- 35-44
- 45-54
- 55-64
- over 65

Marital status

- married
- divorced
- widowed
- single

What is the highest level of education you have obtained?

- some high school
- high school graduate
- some college
- associate's degree
- bachelor degree
- post-graduate degree

Altogether, how many people live in your household, including you?

- 1 (only me)
- 2
- 3 or 4
- 5 or more

How many children under age 15 live in your household?

- 0
- 1
- 2
- 3 or more

How many adults, besides yourself, live in your household?

- 0
- 1
- 2
- 3 or more

Symptom Reporting

In the last three months, since November 1, 2014, which of the following symptoms have you had? Check the box for each one that you had.

- fever
- cough
- sore throat
- headache
- body aches
- fatigue
- runny nose
- stuffed up nose
- vomiting
- diarrhea

- none of these

In the last three months, since November 1, 2014, which of the following symptoms have children in your household had? Check the box for each one that any of the children had.

- fever
- cough
- sore throat
- headache
- body aches
- fatigue
- runny nose
- stuffed up nose
- vomiting
- diarrhea
- none of these

In the last three months, since November 1, 2014, which of the following symptoms have other adults in your household had? Check the box for each one that any of the other adults had.

- fever
- cough
- sore throat
- headache
- body aches
- fatigue
- runny nose
- stuffed up nose
- vomiting
- diarrhea

- none of these

In the last three months, did you try to find information about your symptoms, how to diagnose your condition, or how to treat the symptoms?

- Yes
- No

In the last three months, did you try to find information about the symptoms of others in your household, how to diagnose their condition, or how to treat the symptoms?

- Yes
- No

How did you look for information about these symptoms?

- Open Ended Response

Which of the following sources did you use to look for information about the symptoms you [others in your household] had? Check each one that you used or leave blank if you used none of them.

- Doctor or another healthcare provider
- Book on health or illness
- Health or medicine related website (such as WebMD, Yahoo Health, Mayo Clinic, MedicineNet. etc.)
- Search engine or search assistant (such as Bing, Google, Cortana, Siri, etc.)
- Friend, relative, or someone else I know

Final Diagnosis

Did you see a doctor or another healthcare provider about your symptoms?

- Yes
- No

What diagnosis did the doctor or healthcare provider give you?

- Open Ended Response

Did the other person/people in your household who had symptoms see a doctor or another healthcare provider about their symptoms?

- Yes
- No

What diagnosis/diagnoses did the doctor or healthcare provider give them?

- Open Ended Response

1.4 Information from Healthcare Providers vs. the Internet

Do people search online for health-related information when they have symptoms? Or do they exclusively ask health providers for information? Existing methods from the CDC rely on reporting from health providers to make estimates of flu prevalence. If online searches are to supplant reporting from providers as a method for tracking flu, it must be shown that online searches for symptoms are a common approach to finding illness information.

To answer this question, we looked at differences in the number of respondents who reported seeking information about symptoms from a healthcare provider versus the Internet. 26% reported looking online and asking their healthcare provider for information. We found that only 6% of respondents asked for information from their healthcare provider and did not look online. 33% reported looking for information online and did not ask their provider.

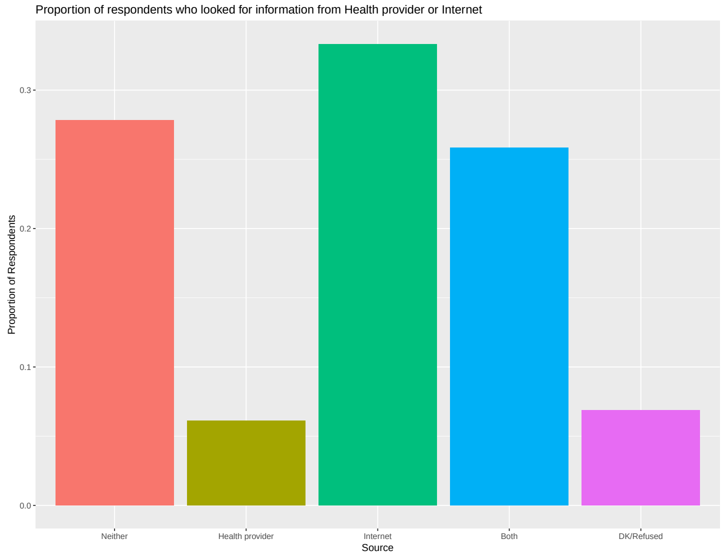

Supplementary Figure 1: Sources of health information. Far more respondents reported looking for information from Internet Sources compared with their healthcare provider

1.5 Query and Web Page Labeling

1.5.1 Overview of Labeling

After isolating the subset of queries and web pages from each survey respondent's web history to be labeled, coders then labeled each candidate query and page, then we joined those labels back to the survey data. This allows us to analyze the effects of different combinations of symptoms and demographics on flu search rates among respondents. This process also reduced the number of queries and webpages to be labeled and resulted in high levels of intercoder agreement and reliability (see supplementary table 7). We

also compute intercoder agreement and reliability scores for the expanded set of flu-like queries from the Bing search engine (see supplementary table 20).

1.5.2 Labeling Scheme

In order to define a flu-related query or web page, we developed a labeling scheme to indicate whether each query or web page was likely related to a flu-like experience, versus experiences related to an array of non-flu infections (including the common cold), news, personal interest, and social trends. In our scheme, we differentiate flu-like experience queries from queries that are unrelated to a flu-like experience but nonetheless contain flu terms. For example, searches like 'fever and cough 5 days', or 'high fever body aches' would qualify as flu-like experience queries. By contrast, 'flu pandemic', 'sore throat obama', or '1918 flu', would qualify as research-oriented and unrelated to a flu experience. Similarly, web pages containing information about flu-like symptoms (e.g. 'fever' and 'cough') would qualify as page visits related to flu-like experiences.

Among queries or pages that convey a flu-like experience, we further identify two forms: experiences of primary flu symptoms, in which one experiences the most common symptoms of flu - fever and cough, and experiences of secondary symptoms, in which one experiences secondary (and less common) symptoms of flu (body aches, diarrhea, vomiting). Queries and web pages that appear consistent with a primary flu-like experience receive an 'A1' label, and queries and pages consistent with secondary flu-like experiences receive a 'B1' label. Queries and pages that appear related to flu but not deemed to be a flu-like experience (journalistic interest or research) receive an 'A2' designation. 'A1' labels capture the primary flu-like experience that we are interested in tracking and are the basis of our subsequent forecasting model. The remainder of the labels serve primarily as gut-checks to validate our data and results.

1.5.3 Codebook: Descriptions of Labels

- A1. Related to experienced ILI – symptoms of canonical flu definition (cough or fever [can be in combination with other symptoms), treatments for flu, generic flu searches/pages (as if from someone searching re how to diagnose or treat).

  Examples: searches - "flu", "cough", "what to do if you have the flu", "productive cough fever body aches", "tamiflu information"; web pages - "Home Remedies for Flu Symptoms (WebMD)"; http://www.rxwiki.com/slideshow/surprising-facts-about-flu/chicken-soup-actually-helps. Keep in mind that pages specifying diagnoses and remedy of canonical flu symptoms go in this category.

- A2. Flu not in the context of experienced probable ILI – trends, prevention (including vaccination), policies, scientific background, etc.

  Examples: Searches - "flu epidemic","flu vaccine 23% effective", "avian flu strain"; web pages - "TABLE. Influenza Vaccines — United States, 2014–15 Influenza Season*, Seasonal Influenza (Flu) CDC"; "Flu Activity & Surveillance, Seasonal Influenza (Flu) , CDC".

- B1. Secondary flu symptoms (generic or as experienced) – sore throat, headache, body aches, fatigue, runny nose, stuffed up nose, vomiting, diarrhea.

  Examples: searches - "sore throat gargle salt water", "sore throat and breast pain", "3 year old runny nose only"; web pages – "Is It a Cold or a Sinus Infection? Symptoms & Treatments (WebMD)", "Sore Throat Home Remedies and Treatments".

- B2. Secondary flu symptoms not in the context of experienced probable ILI

  Examples: searches – "tiger vomits on course", "holiday vomit fest", "pres. obama sore throat"

- C1. Other generic or experienced illness syndromes or symptoms – croup, pneumonia, stomach flu, ear infection, etc.

  Examples: searches – "can adults get croup", "whooping cough", "swollen toe", "cdc ebola"; web pages – `http://www.webmd.com/digestive-disorders/diarrhea-stomach-fl` "Middle ear mucus won't drain. Ear, Nose & Throat Community - Support Group (WebMD)"

- D. Unrelated to human illness

  Examples: searches – "cat cough", "frozen fever" "acdc rock or bust tour", "marisa coughlan feet", "saturday night fever soundtrack"; web pages – "Pflugerville, Texas - Wikipedia, the free encyclopedia"; "Influence and reception of Søren Kierkegaard - Wikipedia, the free encyclopedia"

1.5.4 Labeling Queries and Pages from Respondent Browsing History

Once we had a scheme for labeling queries and pages, we developed a strategy for locating candidate A1 queries from the survey data, given that each user in our panel executed anywhere from dozens to hundreds of queries during the survey period, and it would be inefficient to code every one of them.

To isolate a subsample of queries to label, we started with a simple set of keywords. Using the search history data from our panel, we located a subsample of queries that contained a set of predefined illness-related key words - including terms like: 'sick', 'flu', 'fever', 'cough', 'ache', 'vomiting', 'sore throat'.

For webpages, we selected a set of likely candidate base websites, including: cdc.gov, webmd.com, flu.gov, nih.gov, wikipedia.com, and mayoclinic. We then searched the clickstream for user access to flu related webpages within these domains using the "flu" keyword within the following search structure:

```
where (page_url like '%cdc.gov%' and page_url like '%flu%' )
or (page_url like '%webmd.com%' and page_url like '%flu%')
or (page_url like '%flu.gov%')
or (page_url like '%nih.gov%' and page_url like '%flu%')
or (page_url like '%wiki%' and page_url like '%flu%')
or (page_url like '%mayoclinic%' and page_url like '%flu%')
```

We then tasked trained coders with labeling queries and pages according to our coding scheme (supplementary table 7. In this sample of queries and web pages, we found 21% of respondents made an A1 query or page visit, 14% made an A2 query or page visit, and 9% made a B1 query or page visit. Intercoder reliability on this set was Kappa=.865 for search queries and Kappa=.796 for web pages (see supplementary table 7). In supplementary tables 8 and 9, we display confusion matrices displaying labeling choices from the two trained coders, and specific labels which created the most disagreement.

Supplementary Table 7: Intercoder Reliability - Survey Queries and Pages

	Queries	Web Pages
N	1422	797
Agreement	89%	85.7%
Kappa	0.865	0.796
Z-score	68.5	36.8
p	0.00	0.00
Coders	2	2

Note: two-sided unadjusted p-value

Supplementary Table 8: Intercoder Reliability of Panel Queries - Confusion Matrices

	Queries					
	A1	A2	B1	B2	C1	D
A1	318	2	11	0	17	6
A2	12	120	0	0	0	1
B1	2	0	76	1	25	11
B2	5	0	7	257	5	10
C1	17	0	8	0	283	6
D	3	1	1	0	4	213

Supplementary Table 9: Intercoder Reliability of Panel Web Pages - Confusion Matrices

	Web Pages					
	A1	A2	B1	B2	C1	D
A1	245	31	20	0	1	0
A2	18	263	2	0	28	0
B1	3	1	25	0	1	0
B2	1	0	0	0	0	0
C1	1	0	3	1	105	1
D	1	0	0	0	0	45

The top 10 stemmed words from the respondent set of queries can be observed in supplementary table 10. The reader will note that these queries are not a random sample of queries from the web. Half of these queries came from users selected at the outset because they searched for a flu-related term, while the other half came from users who were selected because they matched the search volume of the first group within a narrow band but did not execute a flu-related query (see description in previous section). As a consequence of this matching process, illness-related words are more common in our sample than in a random draw of queries. We correct for this bias in our statistical models, described in the main text, and in following sections in the SI. In supplementary table 11, the top 10 stemmed words from all queries that matched the A1 criteria as judged by hired coders.

Supplementary Table 10: Top Stemmed Words in All Queries (panel)

word	freq	proportion
flu	257	0.0514
vomit	256	0.0512
cough	163	0.0326
fever	150	0.03
sick	99	0.0198
diarrhea	87	0.0174
swollen	84	0.0168
sore	83	0.0166
girlfriend	70	0.014
throat	70	0.014

Supplementary Table 11: Top Stemmed Words in A1 Queries (panel)

word	freq	proportion
cough	101	0.12
fever	97	0.115
ach	14	0.017
child	14	0.017
grade	11	0.013
infant	11	0.013
low	11	0.013
rash	11	0.013
sick	10	0.012
babi	9	0.011

1.6 Query Data Geographic and Temporal Coverage

Search query data provided in partnership with Bing cover years from 2012 to 2017 for all 50 states plus the District of Columbia. While we are unable to share overall rates of searches from Bing, we can share relative coverage rates for the states and years included. For the state rates, we counted the number of queries in each year, then normalized by population to generate a query rate per capita measure. We then subtracted the state average and divided by the standard error to generate a z-score. We did this separately for the overall query count, flu queries (queries that were flagged as likely related to flu based on our DOC2VEC expansion), and the A1 query count.

For the yearly rates, we calculated the average query count in each year, then subtracted the overall yearly mean and divided by the standard deviation to arrive at the z-score. The figures below show the calculated z-scores by state and year (see supplementary figures 2 and 3).

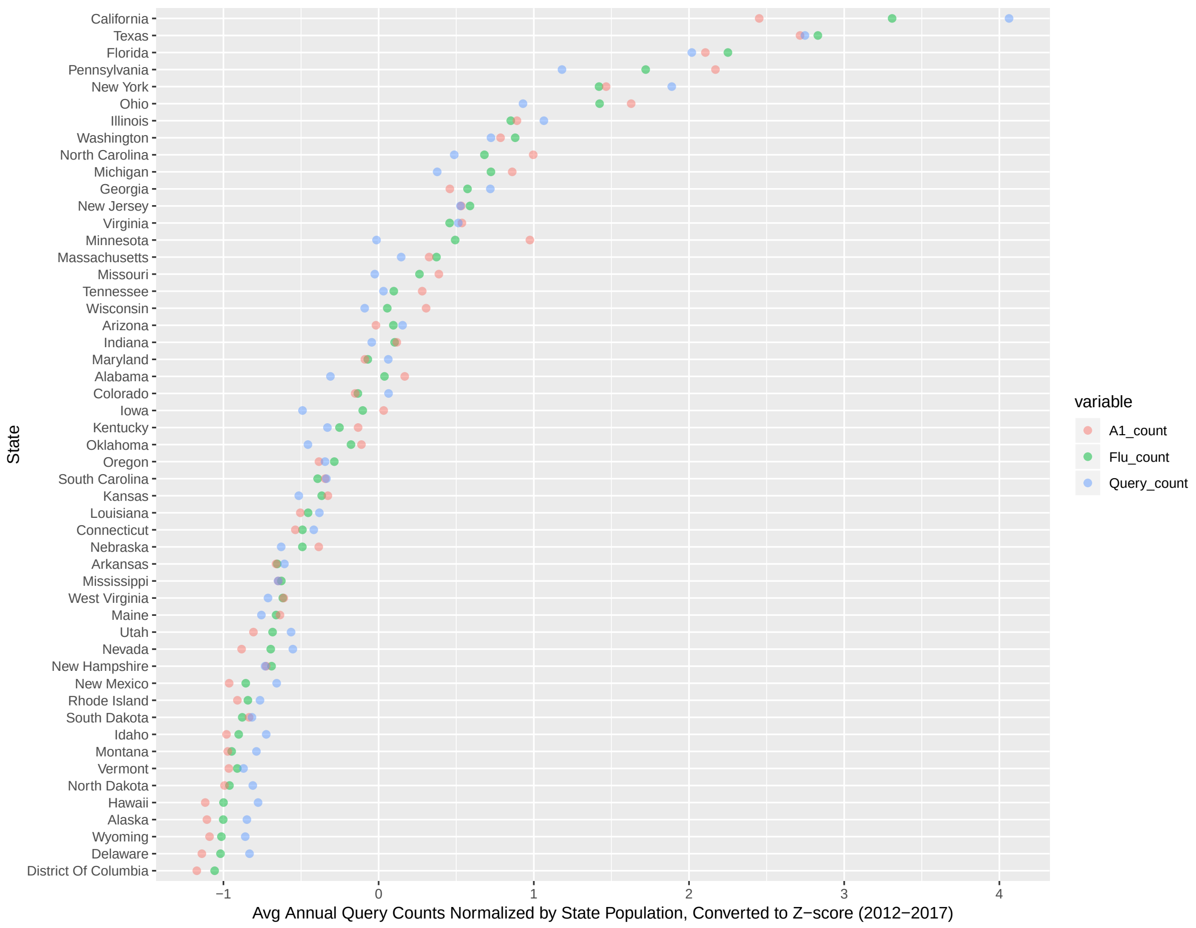

Supplementary Figure 2: State coverage of Bing queries 2012-2017

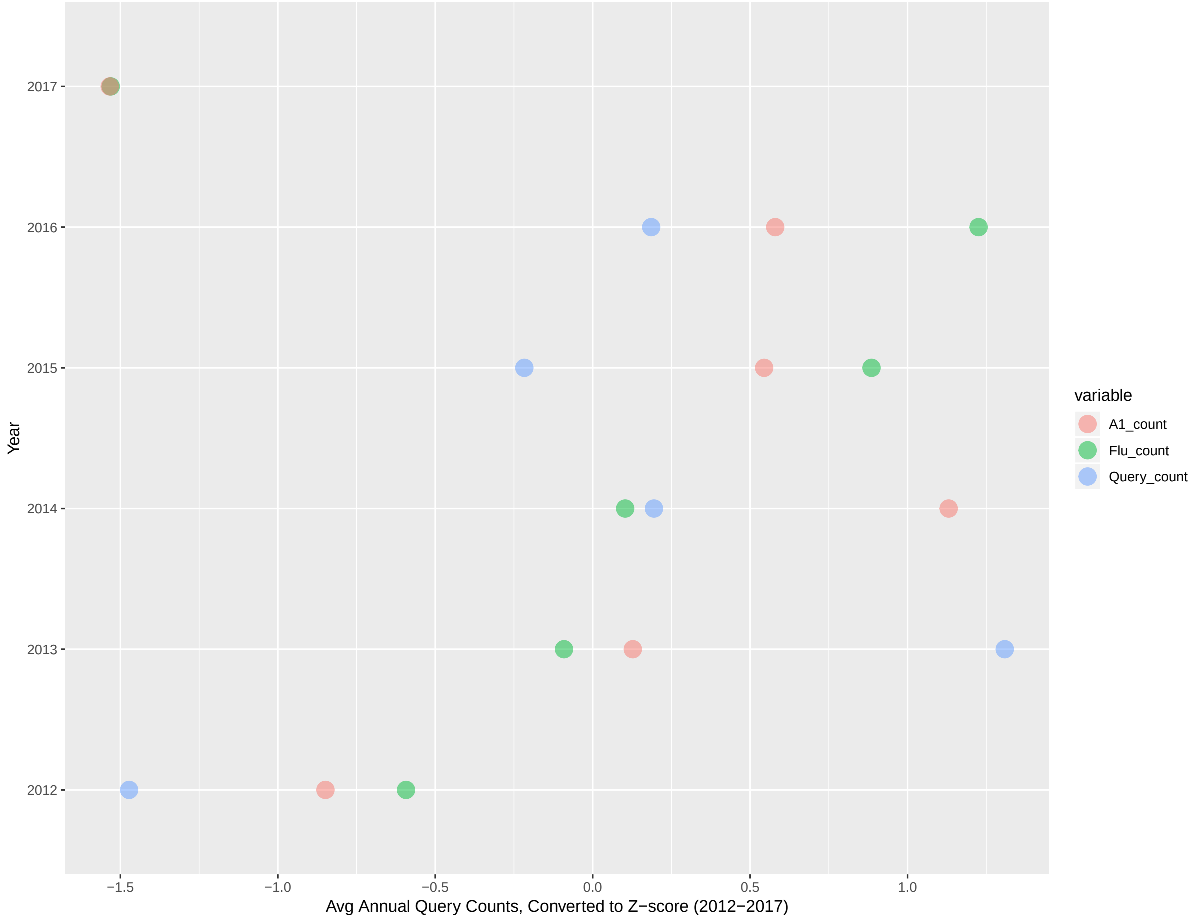

Supplementary Figure 3: Yearly coverage of Bing queries 2012-2017

1.6.1 Predicting Internet Behavior Based on Reported Symptoms

This section provides some additional information on the reported symptoms in our panel and the relationship between these and flu.

Descriptive Relationship

The tables below give the co-occurrence of reported flu symptoms in the household (fever + cough), and queries coded for A1 and A2 in the respondent set of queries. Among respondents reporting flu-like symptoms in the household, roughly 28% made A1 queries or visited relevant pages. Among respondents not reporting flu-like symptoms in the household, roughly 17% made A1 queries or visited pages. In the tables, below we display basic descriptive cross-tabulations of the occurrence of flu symptoms with A1 search (ILI searches) and A2 searches (interest and research queries).

Supplementary Table 12: A1-coded Activities and Flu Symptoms in Household

	not flu	flu	
not A1	354	164	0.32
A1	72	64	0.47
	0.17	0.28	

We construct the same table of A2 searches as supplementary table 12 and compare it to flu symptoms on a subset of the data where respondents had not also executed an A1 query. We expect that since A2 activity is associated with interest or research, it will not be associated with flu symptoms. Here, we see that among respondents reporting flu-like symptoms, roughly 11% made an A2 search, and among respondents not reporting flu-like symptoms, roughly 8% made an A2 search.

Supplementary Table 13: A2 Searches and Flu Symptoms

	not flu	flu	
not A2	375	187	0.33
A2	32	23	0.42
	0.08	0.11	

Regression Model and Case-Control Design

In order to infer the effect of flu-like experience on web activity, we regressed the occurrence of flu-related queries or page visits (A1) on self-reported flu symptoms and demographic characteristics. To infer the effect of the flu on A1 activity, we used a 'classic' or 'cumulative' case-control design. To do so, we first paired positive flu search cases with negative cases, then adjusted for sample differences between the data and the average rate of A1 flu searches from the Bing search engine (1.2e-5). This process allows us to reduce bias induced by the relatively rare occurrence of flu searches in the population. We estimate the search rate using a rare events logistic regression, where $Pr(Y_i = 1|X_i) = \frac{1}{1+e^{-(\beta_0+X_i\beta)}}$. Where β_0 is a constant term, X_i is a k-vector of covariates, and β is a vector of coefficients.

We calculated the relative risk (RR) and risk difference (RD) of search activity given flu symptoms: $RR = Pr(Y = 1|X_1, \tau)/Pr(Y = 1|X_0, \tau)$, $RD = Pr(Y = 1|X_1, \tau) - Pr(Y = 1|X_0, \tau)$ [15]. Where τ is the incidence of A1 searches, X_1 is a k-vector of covariates of a 'treatment' group with flu symptoms and X_0 indicates a k-vector of covariates of a 'control' group lacking flu symptoms. To control for the fact that the population's rate of A1 search differs from our sample, we substitute the constant term in the logistic model for a corrected term that matches the observed rate of flu search in the Bing search engine. This corrected term is calculated as: $B_0 - ln[(\frac{1-\tau}{\tau})(\frac{\bar{y}}{1-\bar{y}})]$, where B_0 is the original constant term, τ is the rate of A1 search in the population, and $\bar{y}$ is the rate of A1 search in the sample. The coefficients remain unbiased. Figure 1 in the main paper plots the expected values of Y, $Pr(Y = 1|X, \tau)$, where household flu is present and absent.

We find a heightened tendency to make both A1 and A2 searches/visits when there is an occurrence of flu symptoms in the household. In figure 1 in the main paper, the means of $= Pr(Y = 1|x_1, \tau)$ and $Pr(Y = 1|x_0, \tau)$ are shown with dark vertical lines. The estimated Risk Ratio (RR) is 1.57 (95% CI = 1.05, 2.34), meaning that those with flu symptoms execute almost 60% more A1 searches compared to those exhibiting no symptoms at all (see supplementary table 14). However, since the base rate is very low, this amounts to a Risk Difference (RD) of about 5.41e-06 (95% CI = 5.57e-07, 1.09e-05), which relates to a very small change in the overall probability of an A1 search given flu symptoms. Nonetheless, the risk difference is statistically significant ($p<.05$).

We also find that searches are noisy indicators for particular subgroups, specifically heavy searchers, women, and parents (see supplementary table 16). We find that A1 searches are correlated with having higher search volumes. People who search more will end up making A1 or A2 searches/visits by chance. Women tend to make more A1 and A2 searches/visits on average, and make many more 'asymptomatic' searches compared to men (where a flu search occurs but no symptoms are reported). Among parents, we find that younger parents are more likely to make A1 searches/visits in the context of a child sick with flu and less likely to make A2 searches/visits. We tested to see if parents of young children are more likely to make A1 searches if their children have flu-like symptoms, but we found no statistically discernible effect.

y = A1 Search	Model 1	Model 2	Model 3	Model 4
(Intercept)	-12.75	-11.51	-12.66	-12.39
	[0.63; 0.00]	[1.12; 0.00]	[0.62; 0.00]	[0.67; 0.00]
Household Flu	0.46			
	[0.21; 0.03]			
Volume	0.15	0.23	0.15	0.17
	[0.07; 0.02]	[0.12; 0.06]	[0.07; 0.03]	[0.08; 0.03]
Female	0.72	0.42	0.74	0.65
	[0.22; 0.00]	[0.37; 0.27]	[0.22; 0.00]	[0.24; 0.01]
Parent	0.33		0.44	
	[0.22; 0.12]		[0.21; 0.03]	
Age	-0.05	-0.42	-0.06	-0.08
	[0.07; 0.46]	[0.17; 0.02]	[0.07; 0.40]	[0.08; 0.29]
Child Flu		0.60		
		[0.33; 0.07]		
Respondent Flu			0.31	
			[0.22; 0.16]	
Spouse Flu				0.06
				[0.29; 0.83]
AIC	645.40	233.19	648.12	523.02
BIC	672.21	249.75	674.93	544.17
Log Likelihood	-316.70	-111.59	-318.06	-256.51
Deviance	633.40	223.19	636.12	513.02
Num. obs.	644	203	644	507

Note that bracketed items below coefficients include [standard error; two-sided unadjusted p-value]

Supplementary Table 14: Rare events logit coefficients estimated using Zelig 5.1.6.1 [7]. Outcome is A1 flu activity.

y=A2	Model 1	Model 2	Model 3	Model 4
(Intercept)	−13.38	−14.55	−13.23	−12.56
	[0.95; 0.00]	[1.93; 0.00]	[0.94; 0.00]	[0.97; 0.00]
Household Flu	0.41			
	[0.31; 0.19]			
Volume	0.19	0.05	0.19	0.08
	[0.10; 0.07]	[0.19; 0.78]	[0.10; 0.07]	[0.11; 0.47]
Female	0.50	0.53	0.52	0.43
	[0.31; 0.11]	[0.60; 0.38]	[0.31; 0.09]	[0.34; 0.21]
Parent	−0.10		0.00	
	[0.33; 0.75]		[0.32; 0.99]	
Age	0.11	0.48	0.10	0.10
	[0.10; 0.27]	[0.28; 0.09]	[0.10; 0.34]	[0.12; 0.41]
Child Flu		1.01		
		[0.55; 0.07]		
Respondent Flu			0.08	
			[0.33; 0.81]	
Spouse Flu				0.36
				[0.39; 0.35]
AIC	373.02	118.71	374.71	304.95
BIC	399.47	134.97	401.16	325.78
Log Likelihood	-180.51	-54.35	-181.35	-147.48
Deviance	361.02	108.71	362.71	294.95
Num. obs.	607	191	607	476

Note that bracketed items below coefficients include [standard error; two-sided unadjusted p-value]

Supplementary Table 15: Rare events logit coefficients estimated using Zelig 5.1.6.1 [7]. Outcome is A2 activity. The sample is a subset of the data in which respondents did not make a concurrent A1 search.

We next re-run our analyses with A2 searches as the outcome variable of interest on a subset of the data where respondents had not executed an A1 query but had executed an A2 query. This serves as a validation check to determine whether A2 or interest-oriented searches are indeed false positive searches. We find no detectable effect of flu symptoms of any household member on the occurrence of A2 research or interest-oriented searches in this subset of the data (see supplementary table 15).

1.6.2 Demographic Groups and Queries

We find that flu-related searches are more likely among particular subgroups, specifically heavy searchers, women, and parents. A1 searches are correlated with having higher search volumes, and people who search more will end up making A1 or A2 searches/visits by chance. Women tend to make more A1 and A2 searches/visits on average, and make

many more 'asymptomatic' searches compared to men (where a flu search occurs but no symptoms are reported).

Among parents, younger parents are more likely to make A1 searches/visits in the context of a child sick with flu and less likely to make A2 searches/visits than older parents. To estimate these effects, we isolated the data to respondents who claimed to be the primary users of their devices (coded as those who claim to use their devices 80% or more of the time relative to spouses and others in the household). We did this to ensure that search activity was limited to the parent and not the child, although the effects are substantial and significant in the broader sample as well. We found that fathers and mothers have different behaviors in reaction to perceived child illness. We find fathers to be much more likely to make A1 searches when their children have an ILI, whereas mothers tend to have a high baseline tendency to make such searches regardless of child illness (see supplementary figure 5). Fathers more than 8 times as many A1 searches when their children exhibit ILI symptoms compared to when they are symptom-free (RR = 8.75, 95% CI = 2.21, 42.36), which amounts to a risk difference of RD=1.95e-05 (95% CI = 5.16-06, 4.53e-05) (see supplementary figure 4). By contrast, we find no such moderating effects for A2 queries - mothers and fathers tend not to exhibit statistically significant differences in A2 search behavior when their children are ill with ILI.

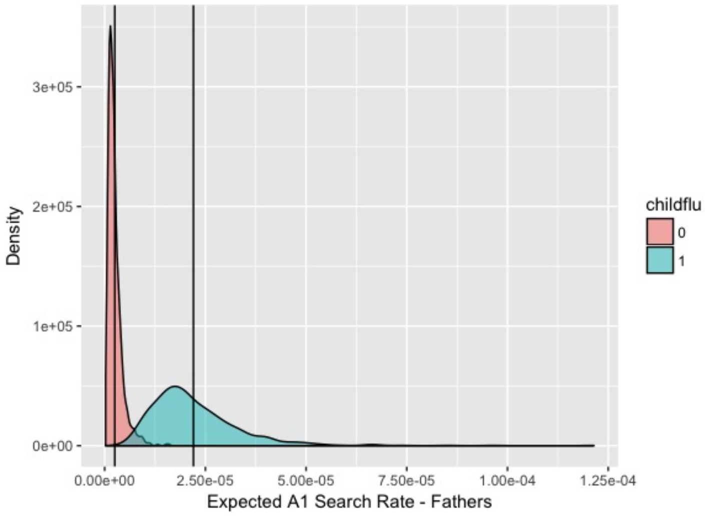

Supplementary Figure 4: Expected father A1 searches when child flu present and absent

Differences in A1 search rates among self-identified racial categories were not significant in our sample. Similarly, differences in A1 search rates among self-reported education levels were not significant. See supplementary table 17 for these results. However, existing surveys using nationally-representative samples suggest that differences Internet usage exist with regard to age, income, education, and other demographic characteristics (`http://www.pewinternet.org/fact-sheet/internet-broadband/`). We control for demographic differences along these lines in our forecasting models.

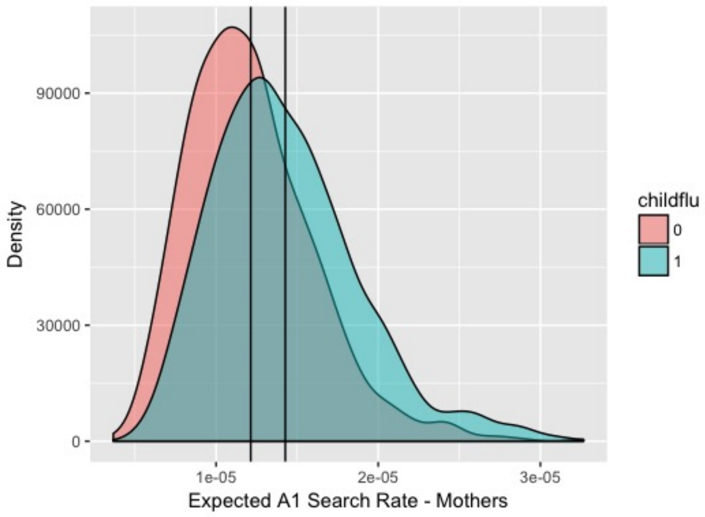

Supplementary Figure 5: Expected mother A1 searches when child flu present and absent

	y = A1 Search	y = A2 Search (subset)
(Intercept)	−12.97	−14.55
	[1.41; 0.00]	[1.97; 0.00]
Child Flu	2.26	1.12
	[0.77; 0.00]	[0.98; 0.25]
Parent gender - Moms	1.73	0.73
	[0.71; 0.01]	[0.91; 0.42]
Volume	0.39	0.06
	[0.14; 0.01]	[0.20; 0.76]
Age	−0.56	0.45
	[0.20; 0.01]	[0.28; 0.11]
Child flu * Parent gender - Moms	−2.10	−0.34
	[0.88; 0.02]	[1.17; 0.77]
AIC	191.93	117.04
BIC	211.09	135.82
Log Likelihood	-89.97	-52.52
Deviance	179.93	105.04
Num. obs.	180	169

Note that bracketed items below coefficients include [standard error; two-sided unadjusted p-value]

Supplementary Table 16: Rare events logit coefficients estimated using Zelig 5.1.6.1 [7]. Outcome is A1 flu activity online.

	Model 1
(Intercept)	−11.42
	[0.25; 0.00]
Household Flu	0.64
	[0.20; 0.00]
Education - No HS	−1.50
	[1.06; 0.16]
Education - Some College	−0.17
	[0.31; 0.60]
Education - Assoc. Degree	0.23
	[0.37; 0.53]
Education - Bach. Degree	−0.21
	[0.30; 0.49]
Education - Grad. Degree	−0.22
	[0.35; 0.52]
Race - Black	−0.07
	[0.36; 0.85]
Race - Native	−0.47
	[1.11; 0.67]
Race - Asian-Pacific	0.06
	[0.39; 0.88]
Race - Hispanic	−0.06
	[0.44; 0.89]
Race - Other	10.85
	[637.46; 0.99]
Race - DK	27.34
	[819.58; 0.97]
AIC	674.68
BIC	732.96
Log Likelihood	-324.34
Deviance	648.68
Num. obs.	654

Note that bracketed items below coefficients include [standard error; two-sided unadjusted p-value]

Supplementary Table 17: Rare events logit coefficients estimated using Zelig 5.1.6.1 [7], including education and race variables

1.6.3 Classifying Flu Cases

While household ILI symptoms may produce A1 activity by an individual, the question remains whether A1 searches and visits provide reliable predictive signals to classify an individual as having ILI symptoms. In order to further validate our coding methodology in a predictive task, we use a machine learning approach to assess the predictive power of the search queries for classifying cases as flu or not. We build basic classification models to classify reported flu occurrence at the household level and the respondent level. In each model, we set aside 30% of the data for validation, leaving the remaining 70% to build and test our models. We use a random forests algorithm to classify each respondent as having an ILI experience or not. We then plot the relative contribution of each variable to the model by examining the increase in accuracy from that variable relative to a random permutation of itself [5]. We calculate these importance scores for each variable then scale them by dividing by the highest score and multiplying by 100 [16, 17] (see supplementary figure 6). Random forest models were parameterized using using 25 bootstrap replications on the training data (all selected mtry=2 for the random forests).

Supplementary Table 18: Confusion Matrix: Household Flu (validation set)

	Reference	
Prediction	$\neg flu$	flu
$\neg flu$	49 (.47)	22 (.21)
flu	14 (.13)	20 (.19)
Sensitivity		0.476
Specificity	0.778	

Household ILI is defined as when a respondent reports one or more of the members of the household have fever and cough. Approximately 40% of respondents reported at least one case of flu in the household. The rate of reported ILI was nearly identical in the training and validation sets - about 41% (n=249) and 40%(n=105), respectively. The random forest model yielded 66.1% accuracy on the training set, and 65.7% accuracy on the validation set (see supplementary table 18)(trained with randomForest version 4.6-12 [20]). The top five predictors of ILI occurrence were age, search volume, being a parent, religion, and education. 'A1' queries were the most predictive of all the coded query types, ranking sixth overall in variable importance measures. Figure 2 displays the variable importance scores from the Household flu model. As above, these scores give the increase in model accuracy relative to a random permutation of each variable.

The household ILI model is able to correctly exclude nearly 80% of non-flu cases in the data (see specificity in supplementary table 18). It was more difficult for the model to correctly include flu cases. The true positive rate (sensitivity) was 47.6%.

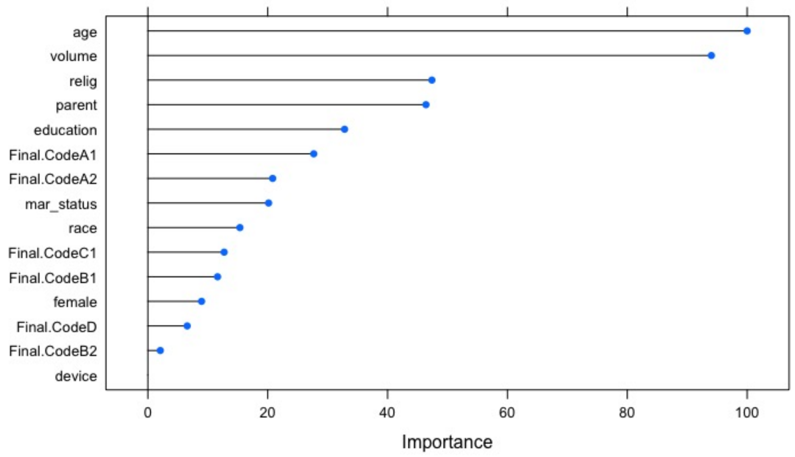

Supplementary Figure 6: Household flu classification model random forest variable importance plot

1.6.4 Robustness Checks: Alternative Panel Models

One potential criticism of our panel analysis is the choice to label a flu-like experience as when an individual reports having both a fever and cough, rather than fever and cough or fever and sore throat. We chose the former operationalization of flu to avoid ruling out (false positive) cases where a respondent reported a sore throat in the context of a separate (non-ILI) illness. Nonetheless, we re-analyzed our panel data with a flu-like experience coded as when a respondent reports fever and cough *or* fever and sore throat.

y = A1 Search	Model 1	Model 2	Model 3	Model 4
(Intercept)	−12.73	−11.38	−12.65	−12.27
	[0.61; 0.00]	[1.10; 0.00]	[0.61; 0.00]	[0.66; 0.00]
Household Flu	0.45			
	[0.21; 0.03]			
Volume	0.18	0.24	0.18	0.18
	[0.07; 0.01]	[0.12; 0.04]	[0.07; 0.01]	[0.08; 0.02]
Female	0.69	0.48	0.70	0.65
	[0.21; 0.00]	[0.37; 0.20]	[0.21; 0.00]	[0.24; 0.01]
Parent	0.25		0.35	
	[0.21; 0.25]		[0.20; 0.08]	
Age	−0.09	−0.48	−0.09	−0.12
	[0.07; 0.23]	[0.17; 0.01]	[0.07; 0.20]	[0.08; 0.14]
Child Flu		0.57		
		[0.33; 0.08]		
Respondent Flu			0.33	
			[0.21; 0.12]	
Spouse Flu				−0.03
				[0.28; 0.93]
AIC	668.37	234.20	670.71	530.73
BIC	695.18	250.77	697.51	551.87
Log Likelihood	-328.18	-112.10	-329.35	-260.36
Deviance	656.37	224.20	658.71	520.73
Num. obs.	644	203	644	507

Note that bracketed items below coefficients include [standard error; two-sided unadjusted p-value]

Supplementary Table 19: Rare events logit coefficients estimated using Zelig 5.1.6.1 [7], with alternative model specifications

The results are similar and consistent with our findings above - rates of flu search are variable across different types of users but are associated with flu symptoms. When flu is reported at the household level, RR = 1.55 (95% CI = 1.042, 2.32), which amounts to a risk difference of RD=5.22e-06 (95% CI = 4.52e-07 1.04e-05), and these differences are statistically significant.

When reexamining the differential effects of mothers and fathers using this alternative measure of flu, when find that fathers have a greater likelihood of flu search in the presence of child flu-like illness compared to mothers—similar to the results presented earlier. In the figure below, we plot this effect. For fathers, we find RR = 7.61 (95% CI = 1.97, 36.76) and RD = 1.60e-5 (95% CI = 3.91e-06 3.70e-05). These effects are slightly milder than those observed above, but we also see somewhat wider variation of the effect under this alternative measurement regime.

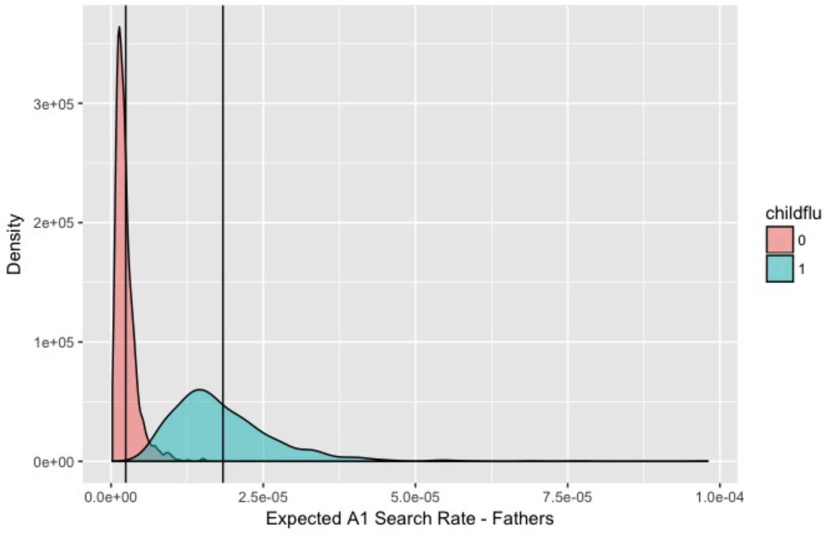

Supplementary Figure 7: Expected effect of child flu on search rates of fathers under alternative flu measure

1.6.5 Takeaways from Panel Analysis

We find evidence to support the claim that search behavior is related to ILI in the household, but also to search volume, age, and gender. Our analysis suggests that a number of different patterns are important. First, not all queries containing the word 'flu' are predictive of ILI experiences. Second, the propensity to conduct searches given ILI experiences (either in the respondent, the household, or in children), is not evenly distributed across demographic groups - as illustrated by the finding that the propensity to search for flu information is correlated with gender, parenthood, and age. This finding poses a challenge for forecasting, because the spread of the flu is also likely to be correlated with demographic features. This analysis raises further questions about methods which do not account for variability across demographic groups when forecasting ILI with web search data.

1.7 ILI Tracking Model

We next describe the multi-step process of building a flu-tracking model using the insights from the case-control model just described. To do so, we first use word-embedding

techniques to generate an expanded list of ‘A1’ search queries based on similar searches in the Bing search engine. We do this since the queries in our relatively small survey are unlikely to capture all the possible queries meeting our our ‘A1’ criteria. Then, we collect a sample of search queries from the Bing search engine and geo-locate them to their origin zip codes. We append census information at the zip code level and use multi-level regression with poststratification to generate a smoothed and re-weighted estimate of observed ‘A1’ activity over the flu season. This estimate is adjusted for demographic features at the zip code level based on census information. This ‘A1’ smoothed estimate is used as the primary predictor of CDC flu prevalance in an ARIMA time-series model.

1.7.1 Selecting and Labeling Additional Bing Queries

Using the labeled subsample of queries matching predefined key words from our panel, we then used an embedding method called 'DOC2VEC' [18] to find queries that are closely related to unseen A1 queries. This allowed us to create an expanded set of likely flu-related queries from the Bing engine for purposes of building our forecasting model. The DOC2VEC method creates document representations based on word embeddings learned from a corpus of text. These word embeddings capture deeper co-occurrence relations that are useful when trying to retrieve similar documents. In this case, our goal was to capture deep co-occurrences with A1 queries from our panel and queries executed on the Bing search engine. We trained a DOC2VEC model on our corpus of A1 labeled queries from our panel containing the word “flu” or “influenza”. Based on this model, we retrieved the 10-nearest neighbors using the Euclidean distance on this representation. We discovered that our approach often captured similarities based on misspelled queries, for example "caugh" and "cough". This information would be lost if instead we trained the word representations on a standard source like Wikipedia.

Based on our DOC2VEC expanded sample of queries, we asked coders to apply the same query labeling scheme as described above. The results of this coding can be seen in supplementary table 20, and in a confusion matrix of coding choices in supplementary table 21. Coder agreement in this expanded set was lower than in the respondent set of queries, likely due to somewhat greater heterogeneity of queries and possibly confusing misspellings. In supplementary table 22 and supplementary table 23 we display the top 10 stemmed queries among all expanded queries and in all expanded queries that were coded as A1, respectively.

Supplementary Table 20: Intercoder Reliability - Expanded Queries from Bing

	Queries
N	1869
Agreement	76%
Kappa	0.57
Z-score	33.2
p	0.00
Coders	2

Note: two-sided unadjusted p-value

Supplementary Table 21: Intercoder Reliability of Expanded Bing Queries - Confusion Matrices

	Web Pages					
	A1	A2	B1	B2	C1	D
A1	824	2	0	0	5	1
A2	348	501	0	0	0	7
B1	4	0	1	0	0	0
B2	4	2	0	0	0	0
C1	61	6	0	0	26	7
D	7	3	0	0	1	59

Supplementary Table 22: Top Stemmed Words in All Queries (Bing)

	word	freq	proportion
1	flu	1,425	0.219
2	shot	292	0.045
3	influenza	283	0.044
4	vaccin	182	0.028
5	symptom	121	0.019
6	stomach	93	0.014
7	cough	89	0.014
8	fever	73	0.011
9	period	58	0.009
10	incub	47	0.007

Supplementary Table 23: Top Stemmed Words in A1 Queries (Bing)

	word	freq	proportion
1	flu	554	0.218
2	cough	132	0.052
3	fever	93	0.037
4	remedi	63	0.025
5	symptom	63	0.025
6	cold	57	0.022
7	influenza	33	0.013
8	headach	28	0.011
9	medicin	28	0.011
10	medic	27	0.011

1.7.2 Accounting for Demographics: Query Smoothing and Re-weighting

If flu search is unevenly spread across subgroups in the population, it then becomes important to control for flu search rates stemming from distinct demographic groups when forecasting flu prevalence within that population. In order to make estimates of flu based on search data, we generate a demographically-weighted estimate of A1 flu search rates over time based on Bing search data. This time-series serves as a demographically-sensitive indicator of underlying rates of flu.

To do so, we collected a long time-series of anonymized web search data from the Bing search engine for a period spanning from early 2011 to late 2016. We geolocated every query to a zip code and classified it as an A1 flu query based on the data coded from the survey panel labeled with the DOC2VEC and human-coder ensemble method. We linked each query to demographic data from the American Community Survey according to its origin zip code.

Based on the demographic information at the zip code level, we used a method called 'multilevel regression with post-stratification' (MRP) to smooth (noise-reduce) and re-weight (reduce bias) against demographic variance in search behaviors [12, 22]. This approach is typically used in survey research to control for sampling bias across demographic groups. We use it to 1) make estimates of flu search behavior across all zip codes at a granular time-scale, and 2) re-weight those estimates by demographic strata shown to be important in our individual-level model.

The MRP method leverages a property of multilevel modeling, "shrinkage", to reduce variance of estimates for units with few observations. In our case, this technique removes noise in flu search rates from less-common geographic areas by bringing them closer to the grand mean of all units. Then, we re-weight all estimates by the prevalence of units with similar demographic characteristics in the census data, we arrive at a population-level estimate of A1 flu-like search propensity.

The MRP model is constructed as follows:

$$Pr(y_i = 1) = logit^{-1}(\beta_0 + \beta_{[it]}^{Income} + \alpha_{j[it]}^{State} + \alpha_{j[it]}^{Education} + \alpha_{j[it]}^{Age} + \alpha_{j[it]}^{Child-per-House} + \alpha_{j[it]}^{Education*Age}$$

Effect are assumed to be drawn from a normal distribution and estimated variance.

Where β_0 is the fixed baseline intercept of the fraction of A1 queries over all queries matching our sample.

$\beta_{[it]}^{\text{Income}}$ is a fixed coefficient corresponding to the median income in that zip code (ACS 2016 one-year data). We chose this predictor because there is evidence that income is associated with an individual's relationship with the Internet (`http://www.pewinternet.org/fact-sheet/internet-broadband/`). We wanted at least one fixed predictor in order to improve the performance of the MRP model [6].

Each of the α variables are random effects and binned by quantiles in order to make them categorical. The random effects are strata to be re-weighted in the MRP setup. This part of the model uses the proportion of residents who had completed a post-high school degree program, age, and the number of children per household.

The variables education, age, and the number of children per house came from the American Community Survey. They were downloaded via the American Community Survey Application Programming Interface (API) using the *acs* package in R (`https://cran.r-project.org/web/packages/acs/acs.pdf`). Education is a binned (by quantile) measure of the proportion of individuals in each zip code who had completed a post-high school degree program, divided by the population of the zip code (these came from the 2014 5-year ACS estimates). Age is a binned (by quantile) measure of the median age in a zip code (from 2014 5-year ACS estimates). Finally, the number of children per house is a binned quantile measure of the average number of children in each household within a zip code (also from the 2014 5-year ACS estimates). These data are available within the replication file.

We use a three-day moving window to construct our MRP estimates (we also tested a 7-day moving window). The results were similar, although a single-day window was noisier. A three-day window gave us enough data to make our estimates while still capturing temporal shifts in search behavior (see [30]). [4]

All MRP models are estimated jointly with no spatial dependence parameters apart from the state indicator in the models using the Lme4 package in R [2]. A prediction is made for each zip code type, scaled by the proportion of the population in the region of interest (US or state), then summed to produce an estimate for the region(s) of interest. For the US as a whole, the prediction for each zipcode type is multiplied by the proportion of the total population it represents, then summed.

1.7.3 More Discussion of Forecasting Results

The following forecasting models are considered in this paper:

- SARIMA-X
  $\phi_p(B)\Phi_P(B^s)Y^{\text{ILI}}(t) = \theta_q(B)\Theta_Q(B^s)\epsilon(t) + I_X\phi_1 X_{\text{mrp}}(t)$
- LASSO-A1
  $Y^{\text{ILI}}(t) = \sum_{i=1}^{p} \theta_i Y^{\text{ILI}}(t-i) + I_X \sum_{j=1}^{m} \phi_j X_j^{\text{A1}}(t) + \epsilon(t)$

for the seasonal ARIMA model with exogenous variables (SARIMA-X) the notation in this paper refers to a ARIMA$(p,d,q) \times (P,D,Q)_s$ model in the Box-Jenkins terminology [4]. We estimate the LASSO-A1 model using a Lasso penalty. This is essentially a class of models referred to as AR-X (Auto-regressive with exogenous). ARGO [29, 30] is an example of this in the context of ILI prediction. The indicator variable I_X refers to the inclusion ($I_X = 1$) or exclusion ($I_X = 0$) of exogenous signals. Pure history based models with $I_X = 0$ serve as a benchmark we compare our models to (no exogenous signals).

We also estimate and test the SARIMA models with the MRP and the normalized total volume of A1 queries as exogenous signals on the States of New Mexico, New York, DC and Delaware.

[4] This window likely induces some ARIMA relationships in the data, but it does so while also capturing a significant amount of the underlying flu rates, as we show in the next section.

1.7.4 State Level Findings

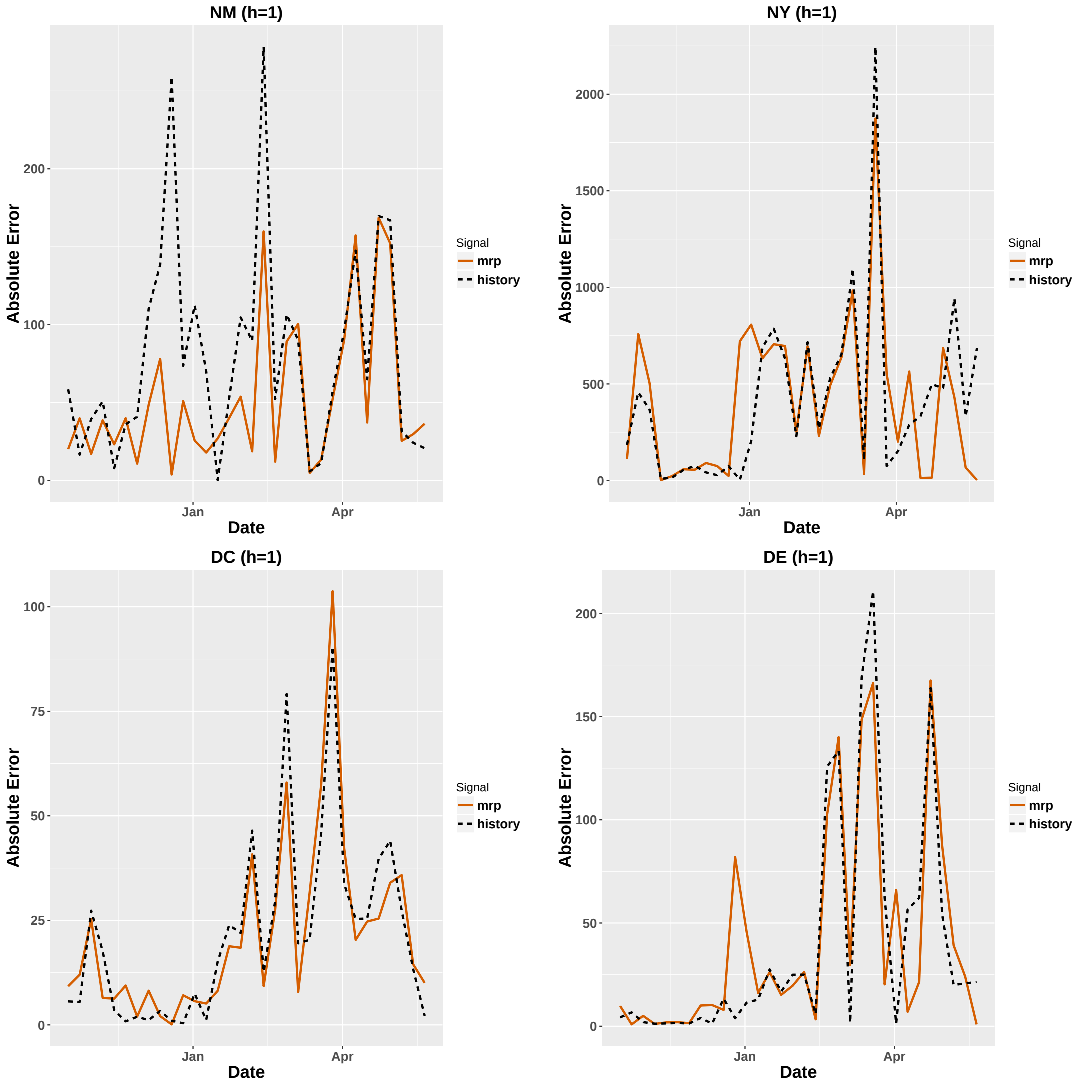

Supplementary Figure 8: a) h=1 indicates week ahead absolute errors for history against the MRP signal for NM, NY, DC and DE.

Supplementary figure 8 shows that incorporating the MRP signal in the SARIMA-X model controls the variance in the prediction error (the absolute errors are less spiky) . These plots visually represent what is presented in Table 3 in the state level RMSE results. As a general approach for predicting time dependent metrics, the inclusion of appropriate exogenous signals such as the MRP signal can have a considerable beneficial impact on the prediction quality.

1.7.5 Software and Data

Census data were collected using the 'acs' package [13] (version 1.2) and the 'noncensus' package [24] (version 0.1).

Data cleaning and analysis were conducted using reshape [26] (0.8.8), reshape2 [26] (1.4.3), ggplot2 [28] (3.1.0), stargazer [14] (5.2.2), effects [10] (4.1-0), Zelig [7] (5.1.6.1), texreg [19] (1.36.23), dplyr [27] (0.78), tidytext [23] (0.2.2), SnowballC [3] (0.6.0), data.table [9] (1.11.8), irr [11] (0.84.1, zoo [31] (1.8-5), lubridate [25] (1.7.4), RColorBrewer [21] (1.1-2), ggthemes [1] (4.2.0), GISTools [8] (0.7-4)

1.8 Notes from main paper

1. Health care provider, health website, search engine, book, friend or none
2. Models are not disaggregated by zipcode, but are partially pooled using hierarchical modeling.

References

1. Arnold, J. B. ggthemes: Extra Themes, Scales and Geoms for 'ggplot2'. *R, ggthemes: Extra Themes, Scales and Geoms for 'ggplot2' (May 12, 2019)* (2019).
2. Bates, D., Mächler, M., Bolker, B. & Walker, S. Fitting Linear Mixed-Effects Models Using lme4. *Journal of Statistical Software* **67,** 1–48 (2015).
3. Bouchet-Valat, M. SnowballC: Snowball Stemmers Based on the C 'libstemmer' UTF-8 Library. *R: SnowballC: Snowball Stemmers Based on the C 'libstemmer' UTF-8 Library (April 1, 2020)* (2020).
4. Box, G. E., Jenkins, G. M., Reinsel, G. C. & Ljung, G. M. *Time series analysis: forecasting and control* (John Wiley & Sons, 2015).
5. Breiman, L. Random forests. *Machine learning* **45,** 5–32 (2001).
6. Buttice, M. K. & Highton, B. How does multilevel regression and poststratification perform with conventional national surveys? *Political Analysis* **21,** 449–467 (2013).
7. Choirat, C. *et al.* Zelig: Everyone's Statistical Software. *R: Zelig: Effect Displays for Linear, Generalized Linear, and Other Models (November 12, 2017)* (2017).
8. Chris Brunsdon, H. C. GISTools: Some further GIS capabilities for R. *R, GISTools: Some further GIS capabilities for R (February 19, 2015)* (2015).
9. Dowle, M. *et al.* data.table: Extension of 'data.frame'. *data.table: Extension of 'data.frame' (July 24, 2020)* (2020).
10. Fox, J. *et al.* effects: Effect Displays for Linear, Generalized Linear, and Other Models. *R: effects: Effect Displays for Linear, Generalized Linear, and Other Models (July 21, 2020)* (2020).

11. Gamer, M., Lemon, J., Fellows, I. & Singh, P. irr: Various Coefficients of Interrater Reliability and Agreement. *R: irr: Various Coefficients of Interrater Reliability and Agreement (January 26, 2019)* (2019).

12. Gelman, A. & Little, T. C. Poststratification into many categories using hierarchical logistic regression. *Survey Methodology* **23,** 127–135 (1997).

13. Glenn, E. H. acs. R: an R package for neighborhood-level data from the US Census. *R: An R Package for Neighborhood-Level Data from the US Census (July 6, 2011)* (2011).

14. Hlavac, M. stargazer: Well-Formatted Regression and Summary Statistics Tables. *R: stargazer: Well-Formatted Regression and Summary Statistics Tables (May 31, 2018)* (2018).

15. King, G. & Zeng, L. Logistic regression in rare events data. *Political analysis* **9,** 137–163 (2001).

16. Kuhn, M. Caret package. *Journal of Statistical Software* **28,** 1–26 (2008).

17. Kuhn, M. & Johnson, K. *Applied predictive modeling* 2013.

18. Le, Q. & Mikolov, T. *Distributed representations of sentences and documents* in *Proceedings of the 31st International Conference on Machine Learning (ICML-14)* (2014), 1188–1196.

19. Leifeld, P. texreg: Conversion of R Regression Output to LaTeX or HTML Tables. *R: texreg: Conversion of R Regression Output to LaTeX or HTML Tables (June 18, 2020)* (2017).

20. Liaw, A. & Wiener, M. Classification and Regression by randomForest. *R News* **2,** 18–22. `https://CRAN.R-project.org/doc/Rnews/` (2002).

21. Neuwirth, E. RColorBrewer: ColorBrewer Palettes. *R, RColorBrewer: ColorBrewer Palettes (February 19, 2015)* (2015).

22. Park, D. K., Gelman, A. & Bafumi, J. Bayesian multilevel estimation with post-stratification: state-level estimates from national polls. *Political Analysis* **12,** 375–385 (2004).

23. Queiroz, G. D. *et al.* tidytext: Text Mining using 'dplyr', 'ggplot2', and Other Tidy Tools. *R: tidytext: Text Mining using 'dplyr', 'ggplot2', and Other Tidy Tools (September 20, 2020)* (2020).

24. Ramey, J. noncensus: U.S. Census Regional and Demographic Data. *R: noncensus: U.S. Census Regional and Demographic Data (May 2, 2014)* (2014).

25. Spinu, V. *et al.* lubridate: Make Dealing with Dates a Little Easier. *R, lubridate: Make Dealing with Dates a Little Easier (June 8, 2020)* (2020).

26. Wickham, H. reshape: Flexibly Reshape Data. *R: reshape: Flexibly restructure and aggregate data usingjust two functions: melt and cast. (Oct 23, 2018)* (2018).

27. Wickham, H., François, R., Henry, L., Muller, K. & RStudio. dplyr: A Grammar of Data Manipulation. *R: dplyr:A Grammar of Data Manipulation (August 12, 2020)* (2020).

28. Wickham, H. *et al.* ggplot2: ate Elegant Data Visualisations Using the Grammar of Graphics. *R: ggplot2: ate Elegant Data Visualisations Using the Grammar of Graphics (June 19, 2020)* (2020).

29. Yang, S., Santillana, M. & Kou, S. C. Accurate estimation of influenza epidemics using Google search data via ARGO. *Proceedings of the National Academy of Sciences* **112,** 14473–14478 (2015).

30. Yang, W., Lipsitch, M. & Shaman, J. Inference of seasonal and pandemic influenza transmission dynamics. *Proceedings of the National Academy of Sciences* **112,** 2723–2728 (2015).

31. Zeileis, A., Grothendieck, G., Ryan, J. A., Ulrich, J. M. & Andrews, F. zoo: S3 Infrastructure for Regular and Irregular Time Series (Z's Ordered Observations). *R, zoo: S3 Infrastructure for Regular and Irregular Time Series (Z's Ordered Observations) (May 2, 2020)* (2020).
