# Supplementary material for: Survey data and human computation for improved flu tracking
Source: Nat Commun. 2021 Jan 8;12:194. doi: 10.1038/s41467-020-20206-z (PMC7794445; doi:10.1038/s41467-020-20206-z)
Supplement: Supplementary file 3 — Reporting Summary [file 41467_2020_20206_MOESM3_ESM.pdf]

nature research

Corresponding author(s): Stefan Wojcik

Last updated by author(s): Nov 6, 2020

Reporting Summary

Nature Research wishes to improve the reproducibility of the work that we publish. This form provides structure for consistency and transparency in reporting. For further information on Nature Research policies, see our Editorial Policies and the Editorial Policy Checklist.

Statistics

For all statistical analyses, confirm that the following items are present in the figure legend, table legend, main text, or Methods section.

n/a	Confirmed	
☐	☒	The exact sample size (*n*) for each experimental group/condition, given as a discrete number and unit of measurement
☐	☒	A statement on whether measurements were taken from distinct samples or whether the same sample was measured repeatedly
☐	☒	The statistical test(s) used AND whether they are one- or two-sided<br>*Only common tests should be described solely by name; describe more complex techniques in the Methods section.*
☐	☒	A description of all covariates tested
☐	☒	A description of any assumptions or corrections, such as tests of normality and adjustment for multiple comparisons
☐	☒	A full description of the statistical parameters including central tendency (e.g. means) or other basic estimates (e.g. regression coefficient) AND variation (e.g. standard deviation) or associated estimates of uncertainty (e.g. confidence intervals)
☐	☒	For null hypothesis testing, the test statistic (e.g. *F*, *t*, *r*) with confidence intervals, effect sizes, degrees of freedom and *P* value noted<br>*Give P values as exact values whenever suitable.*
☐	☒	For Bayesian analysis, information on the choice of priors and Markov chain Monte Carlo settings
☐	☒	For hierarchical and complex designs, identification of the appropriate level for tests and full reporting of outcomes
☐	☒	Estimates of effect sizes (e.g. Cohen's *d*, Pearson's *r*), indicating how they were calculated

Our web collection on statistics for biologists contains articles on many of the points above.

Software and code

Policy information about availability of computer code

Data collection	Census data were collected using the ‘acs’ package (version 1.2) and the ‘noncensus’ package (version 0.1).
Data analysis	Data cleaning and analysis were conducted using reshape (0.8.8), reshape2 (1.4.3), ggplot2 (3.1.0), stargazer (5.2.2), effects (4.1-0), Zelig (5.1.6.1), texreg (1.36.23), dplyr (0.78), tidytext (0.2.2), SnowballC (0.6.0), data.table (1.11.8), irr (0.84.1, zoo (1.8-5), lubridate (1.7.4), RColorBrewer (1.1-2), ggthemes (4.2.0), GISTools (0.7-4)

For manuscripts utilizing custom algorithms or software that are central to the research but not yet described in published literature, software must be made available to editors and reviewers. We strongly encourage code deposition in a community repository (e.g. GitHub). See the Nature Research guidelines for submitting code & software for further information.

Data

Policy information about availability of data

All manuscripts must include a data availability statement. This statement should provide the following information, where applicable:

- Accession codes, unique identifiers, or web links for publicly available datasets
- A list of figures that have associated raw data
- A description of any restrictions on data availability

main_flu_dat.rds: survey data, each row is a survey response, columns describe each respondent, id column is RID
queries.rds: query data from respondents, each row is a query, id column is QID2
pages.rds: page visits from respondents, each row is a page visit, id column is QID2
expanded_queries.rds: queries expanded using Doc2Vec technique, each row is a query, coded by assistants
expanded_queries_final.rds: final set of coded queries, with a single code
tau.rds: the estimated base rate of flu search as a proportion of all searches

panel_demographics.csv: more limited survey data with searches, demographics, and symptoms of users
main_flu_dat_sorethroat.rds: survey data with searches, demographics, and symptoms of users (using alternative definition of flu)
panel_demographics_sorethroat.csv:more limited survey data with searches, demographics, and symptoms of users (using alternative definition of flu
zipcodeCensusData_v2.rds: Zipcode level demographic data from the 2014 5-year American Community Survey (ACS) estimates, including education, age, and the number of children per house, collected using the 'acs' package listed in software.
mrp_example_data.csv: Example search query data and including merged ACS data from reshuffled
NMRaw.csv: the number of positive influenza swabs in New Mexico 2012-2017 based on the weekly Influenza reports (https://github.com/thcoleman/Flu-data-scraping)
NYRaw.csv: the number of positive influenza swabs in New York 2012-2017 based on the weekly influenza reports (https://github.com/thcoleman/Flu-data-scraping)
DCRaw.csv: the number of positive influenza swabs in District of Columbia 2012-2017 based on the weekly influenza reports (https://github.com/thcoleman/Flu-data-scraping)
DERaw.csv: the number of positive influenza swabs in Delaware 2012-2017 based on the weekly influenza reports (https://github.com/thcoleman/Flu-data-scraping)
USFlu.csv: percent of ILI cases in the US 2012-2017 from the CDC (https://www.cdc.gov/flu/weekly/index.htm)
Data restrictions: The raw search query data used for the MRP smoothing cannot be shared publicly as it contains potential PII and is proprietary. Therefore, we only include a shuffled snippet of the data used for the MRP smoothing to allow for conceptual replication of the process. The rest of the code and data are available publicly at https://github.com/stefanjwojcik/ms_flu.

Field-specific reporting

Please select the one below that is the best fit for your research. If you are not sure, read the appropriate sections before making your selection.

☐ Life sciences ☒ Behavioural & social sciences ☐ Ecological, evolutionary & environmental sciences

For a reference copy of the document with all sections, see nature.com/documents/nr-reporting-summary-flat.pdf

Behavioural & social sciences study design

All studies must disclose on these points even when the disclosure is negative.

Study description

This quantitative study uses profesionally-procured survey data from a vendor (Luth Research), and search query data from a popular search engine.

Research sample

After matching (see below) we paired 262 respondents who undertook a flu search with 382 who were drawn randomly from the 20,000 individual sample from Luth Research. This procedure was chosen to be able to compare those who undertook a search with those who did not. From this sample:
The mean age was 45.91 (sd = 14.22);
61% of respondents were female;
336 were employed full-time, 75 were employed part-time, 98 were retired, 28 were students, 43 were looking for work, 52 were unemployed, and 23 listed "other";
334 were married, 215 were single and never married, 73 were divorced, 28 were widowed, and 5 were separated;
About 31% of respondents were parents;
The mean number of children 0-10 was 0.88 (sd = 1.02), the mean number of children 11-17 was 0.74 (sd = 0.80), and the mean number of adult children in the household was 0.41 (sd = 0.60).
16 did not complete high school, 107 had a high school diploma or equivalent, 151 had some college, 65 had an associate's degree, 211 had a bachelor's degree, and 105 had a graduate or professional degree.
While this matched sample does not reflect the proportions in the U.S. Census, the most important factor in the MRP procedure utilized in this paper is that we had enough variety in the sample demographics that we can reasonably estimate the prevalence across different groups. This was clearly the case for the demographics of this sample.

Sampling strategy

We partnered with a survey vendor, Luth Research, who works periodically with Microsoft Research conducting opinion research. Their ongoing panel includes approximately 20,000 individuals with personal computers. Luth research recruits users based on a quota system to construct a panel that is representative of Internet-connected US adults. All individuals agreed to participate in marketing research in return for monetary compensation. This agreement included installing a program to track their web browsing and search activities while also responding to questionnaires.
We selected two subsets from the full 20,000 panel to participate in our research. One set of participants met the following criteria: 1) they had executed queries in any search engine (incl. Bing, Google, Yahoo), 2) these queries included predefined flu-related keywords (e.g. 'flu', 'fever', 'influenza', 'swollen', 'cough', 'pneumonia', 'sore throat'), orthese users visited flu-related URLs (e.g. on WebMD, CDC, Wikipedia). The second group was a comparison group that did not execute a flu-related query or visit a flu-related web site.Using the 2014-2015 flu season we collected all query data starting November 2014 through February 2015 to use as a benchmark in matching users. Individuals in our sample who made flu-related searches had, on average, much higher search volumes than those who did not. To account for this uneven mixture, we matched users on their search volume quantiles. The dataset was set up into 7 quantiles based on the level of search volumes and non-flu-search cases were paired with flu search cases on a 4:1 basis. In total we had 1,180 in the search group and 4,000 in the non-search group. After this matching, there was no statistically significant difference in mean search volume between the groups (p = 0.77). From these individuals, we collected demographic information and collected survey results from a total of 654 individuals, of which 10 did not have any reported search volume in the sample period (see table 5). This left us with 262 who had searched a flu-related keyword or site and 382 who did not (omitting the 10 with zero search volume). This sample size with a handful of predictors (~7) allows us to detect effects well below .1 with a power of .8

Data collection

The study was observational, so participants were not given experimental treatments of any kind.

Timing	We fielded our flu survey in the spring of 2015. We fielded a first wave of the survey from March 19th to March 27th 2015, then followed up with a second wave in the field from April 27th to April 31.
Data exclusions	Ten participants who did not execute any searches during the survey period were excluded from the analysis, as this study is about search queries.
Non-participation	We only included respondents who agreed to participate in the survey. Complete data on invitees and completes are provided in the supporting information. In total we invited 1,180 from the flu-search group and 4,000 in the non-search group. From these individuals,<br>we collected demographic information and collected survey results from a total of 654 individuals.
Randomization	This was an observational, rather than experimental, design.

Reporting for specific materials, systems and methods

We require information from authors about some types of materials, experimental systems and methods used in many studies. Here, indicate whether each material, system or method listed is relevant to your study. If you are not sure if a list item applies to your research, read the appropriate section before selecting a response.

Materials & experimental systems

n/a	Involved in the study	
☒	☐	Antibodies
☒	☐	Eukaryotic cell lines
☒	☐	Palaeontology and archaeology
☒	☐	Animals and other organisms
☐	☒	Human research participants
☒	☐	Clinical data
☒	☐	Dual use research of concern

Methods

n/a	Involved in the study	
☒	☐	ChIP-seq
☒	☐	Flow cytometry
☒	☐	MRI-based neuroimaging

Human research participants

Policy information about studies involving human research participants

Population characteristics	The mean age was 45.91 (sd = 14.22);<br>61% of respondents were female;<br>336 were employed full-time, 75 were employed part-time, 98 were retired, 28 were students, 43 were looking for work, 52 were unemployed, and 23 listed “other”;<br>334 were married, 215 were single and never married, 73 were divorced, 28 were widowed, and 5 were separated;<br>About 31% of respondents were parents;<br>The mean number of children 0-10 was 0.88 (sd = 1.02), the mean number of children 11-17 was 0.74 (sd = 0.80), and the mean number of adult children in the household was 0.41 (sd = 0.60).<br>16 did not complete high school, 107 had a high school diploma or equivalent, 151 had some college, 65 had an associate’s degree, 211 had a bachelor’s degree, and 105 had a graduate or professional degree.<br>24.5% of respondents reported flu-like symptoms
Recruitment	Luth Research recruits for PanelOne from its existing SurveySavvy research panel of more than 3-million respondents. SurveySavvy is a double opt-in panel of survey taking respondents who register with an interest to participate in market research. SurveySavvy's growth is primarily referral based - leveraging Luth Research's patented three tier referral process. Luth Research adheres to a strict privacy policy governing the use of all data collected, including compliance with ISO standards for data security and HIPPA regulations.<br>Being part of both Panel One and taking part in surveys each provide monetary compensation. User participants know and willingly agree to being tracked online and take surveys. This can create biases in the population based on those willing to share search information for compensation and who is willing to take surveys in return for compensation. Participants are aware of being part of SurveySavvy and PanelOne but do not know who requests surveys (in this case unaware that microsoft corporation sponsored the survey research).
Ethics oversight	Northeastern University # 18-07-03

Note that full information on the approval of the study protocol must also be provided in the manuscript.
